# Exploring Triphala as a Biocompatible Alternative to Chemical Denture Cleansers: Effects on Flexural Strength and Surface Roughness of Polymethyl Methacrylate (PMMA) and Flexible Polymers

**DOI:** 10.7759/cureus.99494

**Published:** 2025-12-17

**Authors:** Lokesh B Kanchan, Rakshith Guru, Santhosh Sathyanarayan, Dwarakananda N Bukya, Pavan Kulkarni, Rachana P Hiremath

**Affiliations:** 1 Prosthodontics, Crown and Bridge, ESIC Dental College, Kalaburagi, IND

**Keywords:** denture cleanser, flexible denture, flexural strength, pmma, surface roughness, triphala

## Abstract

Purpose: The longevity and functional performance of denture base materials can be significantly influenced by routine hygiene maintenance. Natural alternatives such as Triphala may offer accessible and cost-effective cleansing solutions, especially in settings where commercial denture cleansers are less available. This in vitro study aimed to evaluate and compare the effects of Triphala and a commercial cleanser (Clinsodent) on the flexural strength and surface roughness of flexible and conventional polymethyl methacrylate (PMMA) denture base materials after prolonged use.

Study design: An in vitro comparative experimental study conducted under standardized laboratory conditions.

Materials and methods: A total of 120 denture base specimens (n=30 per group) were fabricated using two materials: flexible nylon-based polymer and heat-cured PMMA. The specimens were divided into four groups based on material type and cleansing agent used. All specimens were subjected to overnight immersion in their respective solutions for 120 consecutive days. Flexural strength was measured using the three-point bending method, while surface roughness was evaluated using a contact profilometer. Statistical analysis was performed using one-way ANOVA and Tukey’s post hoc test (p<0.05).

Results: PMMA specimens exhibited significantly higher flexural strength compared to flexible materials (p<0.05). Triphala-treated samples showed reduced surface roughness in both materials compared to those treated with Clinsodent. Group 2 (PMMA + Triphala) demonstrated the highest flexural strength (91.79±0.04 MPa), while Group 3 (flexible + Clinsodent) recorded the lowest (28.52±0.16 MPa). Surface roughness was lowest in Group 2 (0.23±0.02 μm) and highest in Group 3 (1.71±0.03 μm).

Conclusion: Triphala may be a promising natural alternative to chemical denture cleansers, with favorable effects on surface roughness and acceptable mechanical performance. However, long-term clinical studies are warranted to validate these findings.

## Introduction

Maintaining denture hygiene is crucial for ensuring the functional longevity of prostheses and preventing oral diseases such as denture stomatitis. The accumulation of microbial biofilms on denture surfaces, particularly Candida albicans, contributes to mucosal inflammation, halitosis, and deterioration of denture materials [1-3]. Poor cleansing practices promote biofilm maturation, discoloration, and mechanical degradation, ultimately compromising oral health and prosthesis performance [3].

Mechanical cleaning methods, such as brushing, are effective in plaque removal but may induce surface abrasions and microcracks, increasing the risk of microbial adherence [4]. Therefore, chemical denture cleansers are often preferred for their convenience and antimicrobial effectiveness. However, most commercial cleansers contain perborates, peroxides, or hypochlorites, which, with repeated exposure, can negatively affect surface integrity, color stability, and flexural properties of denture base resins [5-7]. Additionally, factors such as cost, accessibility, and mucosal irritation can limit their widespread use, particularly among elderly or institutionalized populations [6,7].

Recently, herbal and plant-based alternatives have gained attention as safe, biocompatible, and cost-effective substitutes for chemical cleansers. Among these, Triphala, a traditional Ayurvedic formulation composed of Terminalia chebula, Terminalia bellirica, and Emblica officinalis, has demonstrated potent antimicrobial, antioxidant, and anti-inflammatory effects [8-10]. Several in vitro studies have confirmed its inhibitory action against Candida albicans and Streptococcus mutans, suggesting potential use as a denture cleanser [10,11]. The polyphenolic compounds in Triphala, such as gallic acid, ellagic acid, and tannins, exhibit chelating and cleansing properties that can effectively remove biofilm and organic debris without inducing surface damage [9,12].

Despite its established antimicrobial potential, limited research has explored the effects of Triphala on the mechanical and surface properties of denture base materials following prolonged exposure. Polymethyl methacrylate (PMMA) remains the gold standard for denture fabrication due to its aesthetics, stability, and ease of repair, though its water sorption and sensitivity to chemical degradation may compromise its durability [13,14]. Flexible nylon-based polymers offer enhanced comfort and aesthetics but exhibit lower flexural strength and greater susceptibility to surface roughening and plasticizer leaching [14,15].

Hence, understanding how Triphala affects the flexural strength and surface roughness of PMMA and flexible denture base materials under long-term immersion conditions is clinically relevant. The present study aims to compare the influence of Triphala and a commercial denture cleanser (Clinsodent) on these key physical properties, simulating the effect of 120 days of overnight cleansing.

## Materials and methods

A total of 120 standardized rectangular bar specimens were fabricated in accordance with ISO 20795-1 standards, measuring 65x10x3 mm. Sixty specimens were made using heat-cured PMMA (Lucitone 199® denture resin, Dentsply Sirona, USA), and 60 were fabricated from flexible thermoplastic nylon polymer (Valplast®, Valplast International Corp., New York, United States). Heat-cured PMMA (Lucitone 199® denture resin, Dentsply Sirona, USA) was processed using a standard long curing cycle: polymer-monomer ratio 3:1, dough-stage packing, curing at 74°C for 90 minutes followed by 100°C for 30 minutes, and bench cooling for one hour before deflasking. Flexible nylon specimens (Valplast®, Valplast International Corp., New York, United States) were fabricated using an injection-molding system at 280°C±10°C, 6 bar pressure, and 15 s injection time, followed by ambient cooling. All specimens were finished and polished using a standardized protocol consisting of sequential wet grinding with 240-, 400-, 600-, and 800-grit silicon carbide papers, followed by pumice and rouge polishing on a lathe wheel. 

Sample size estimation

A priori sample size calculation was performed using data from Tripathi et al. [16]. Using a conservative assumed standard deviation (SD) of 0.20 µm, a detectable difference of 0.20 µm, a two-sided α of 0.05, and 90% power, the statistical requirement was 22 specimens per group, which increased to 26 per group after applying a 15% buffer for potential specimen loss. Although the reported SDs in the referenced article would mathematically justify a smaller sample size, a more robust approach was adopted. Therefore, 30 specimens per group were included, resulting in a total of 120 specimens, ensuring adequate power and methodological rigor.

Inclusion criteria

Specimens fabricated from heat-cured PMMA denture base resin and flexible denture base resin with standardized dimensions were included. Specimens meeting ISO-specified dimensional accuracy and surface integrity requirements were included. Specimens free from visible defects such as porosities, cracks, warpage, or incomplete polymerization were included. Specimens processed, finished, and polished using standardized protocols to ensure uniformity across all experimental groups were included.

Exclusion criteria

Specimens presenting surface imperfections, internal voids, or fabrication defects detected during visual inspection or microscopic evaluation were excluded. Specimens with dimensional deviations beyond the acceptable tolerance limits were excluded. Specimens damaged during fabrication, handling, storage, or immersion procedures were excluded. Specimens contaminated or exposed to any chemical agent other than the designated cleanser solutions were excluded.

Specimens were randomly divided into four groups (n=30) using the lottery method: Group 1 (PMMA + Clinsodent), Group 2 (PMMA + Triphala), Group 3 (flexible polymer + Clinsodent), and Group 4 (flexible polymer + Triphala).

Preparation of Triphala solution

**Figure 1:**
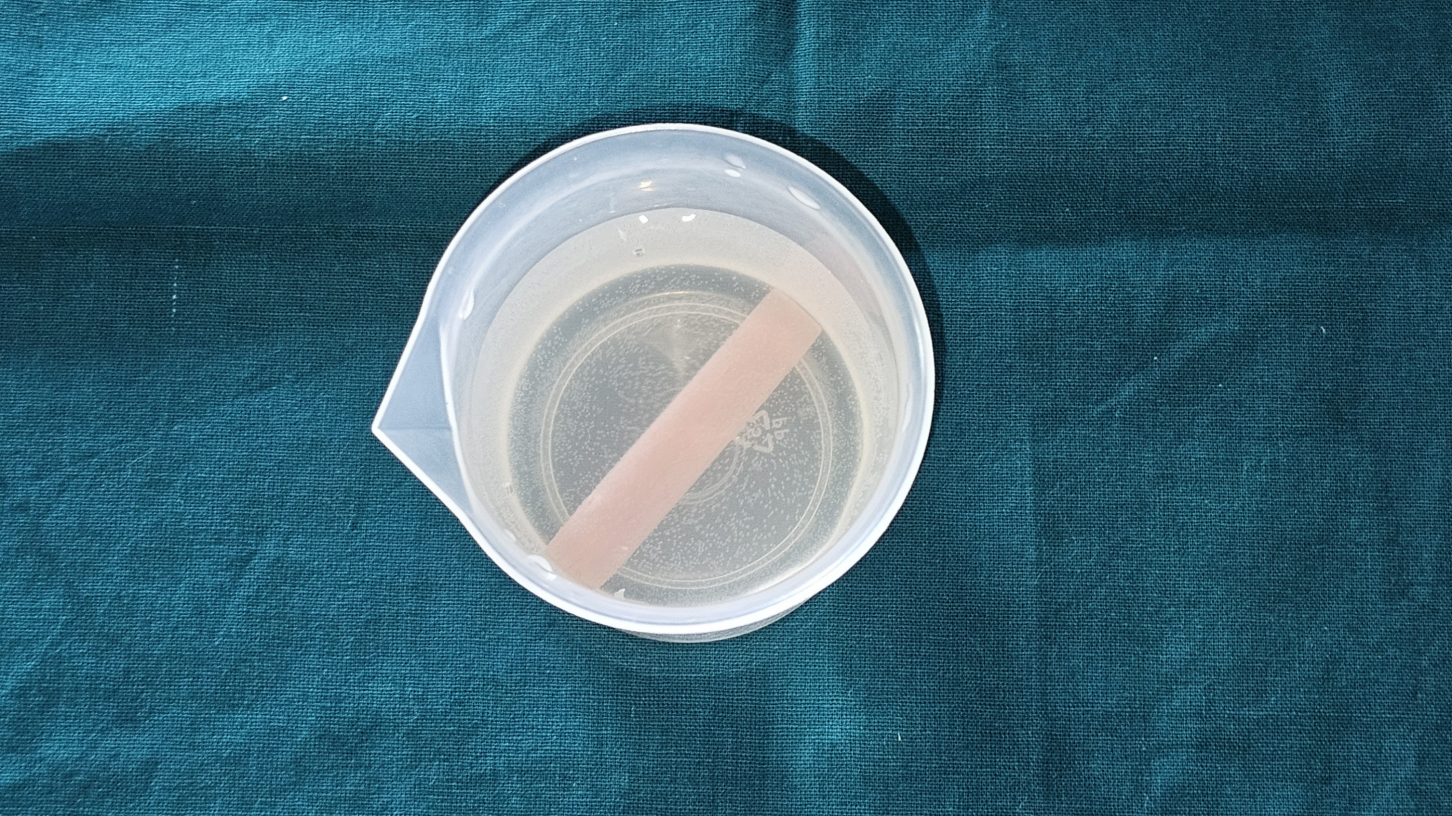
Specimen immersed in Triphala solution

Triphala powder (Triphala Churna, Patanjali Ayurved Limited, Uttarakhand, India) was weighed to prepare a 15% w/v solution. For every 100 mL of distilled water, 15 g of Triphala powder was measured and transferred into a clean beaker. The mixture was stirred continuously using a magnetic stirrer until the powder was completely dissolved and a homogeneous solution was obtained. The solution was freshly prepared daily to maintain its stability and efficacy. All specimens were immersed in this freshly prepared solution according to the study protocol (Figure 1).

**Figure 2:**
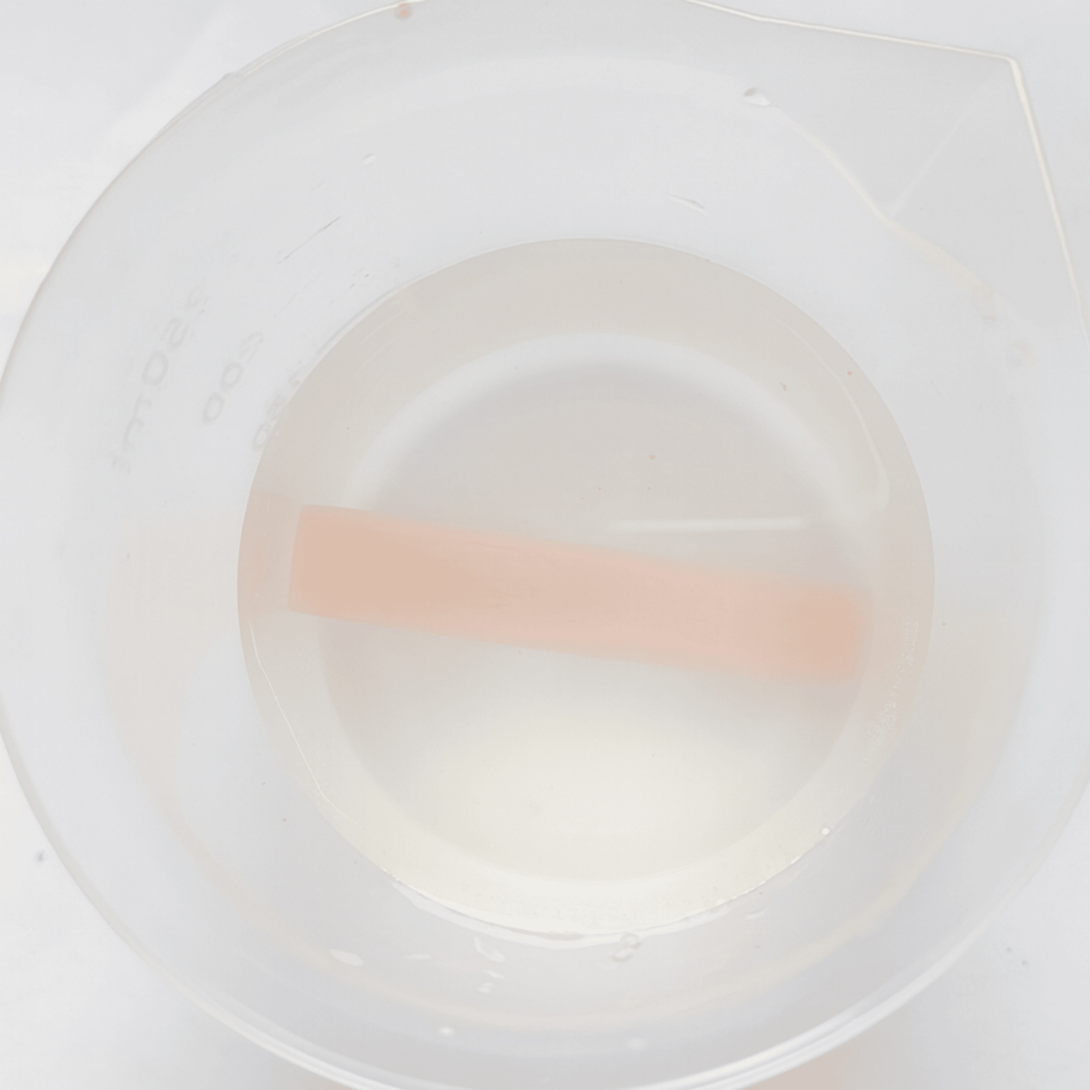
Specimen immersed in Clinsodent solution

Clinsodent (ICPA Health Products Ltd, Mumbai, India), which is a sodium perborate-based denture cleanser, was prepared as per the manufacturer's instructions. All specimens were immersed overnight (eight hours) in their respective solutions for 120 days to simulate typical nightly denture use and approximate long-term effects in a controlled in vitro setting (Figure 2).

Post-immersion, specimens were rinsed, air-dried, and stored under standard conditions, including controlled temperature (37°C±1°C), humidity, and consistent handling, in accordance with ISO 20795-1.

**Figure 3:**
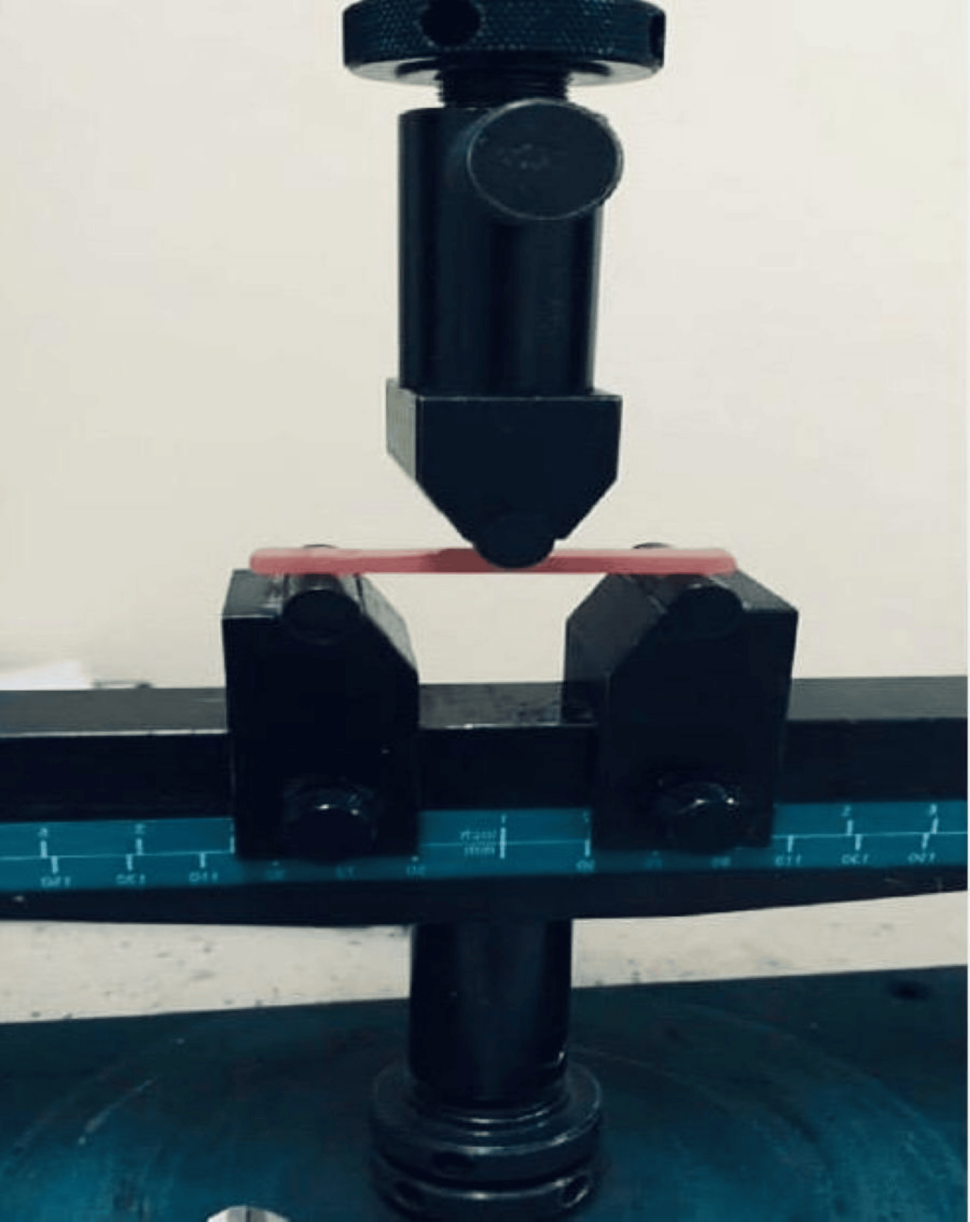
Flexural strength measurement using a universal testing machine

Flexural strength was measured using a universal testing machine via the three-point bending method (ADMET, Inc., Massachusetts, USA) at a span length of 50 mm and a crosshead speed of 5 mm/min (Figure 3).

Surface roughness was measured with a contact profilometer (Mitutoyo Corporation, Kanagawa, Japan). Each specimen was scanned at three standardized locations: the central area and two lateral areas, 5 mm from each edge, along the length of the polished surface. At each location, the profilometer tip traced a 5 mm linear path, and the mean Ra value of the three scans was recorded. A surface roughness (Ra) change greater than 0.2 µm was considered clinically relevant, as increases beyond this threshold can enhance plaque retention and become perceptible to patients [4]. Likewise, a flexural strength value below 65 MPa was regarded as clinically significant, since reductions beyond this limit may compromise the functional durability expected for denture base materials in accordance with ISO 20795-1 standards [17]. All instruments, including the profilometer and load cell, were calibrated and zeroed prior to measurements.

Statistical analysis was carried out using SPSS software version 25.0 (IBM Corp., Armonk, NY) to evaluate differences in flexural strength and surface roughness across the four groups. Descriptive statistics (mean, SD, and standard error (SE)) were computed for all variables. The Shapiro-Wilk test was used to assess the normality of the data, and all datasets met the assumption of normal distribution (p>0.05). Homogeneity of variances was examined using Levene’s test; however, significant results (p<0.001) indicated unequal variances among groups. In view of this variance heterogeneity, Welch’s one-way ANOVA was selected as the primary inferential test to compare group means. When significant omnibus differences were detected, Games-Howell post hoc tests were employed to determine specific pairwise differences. The significance level for all statistical tests was set at p<0.05.

**Figure 4:**
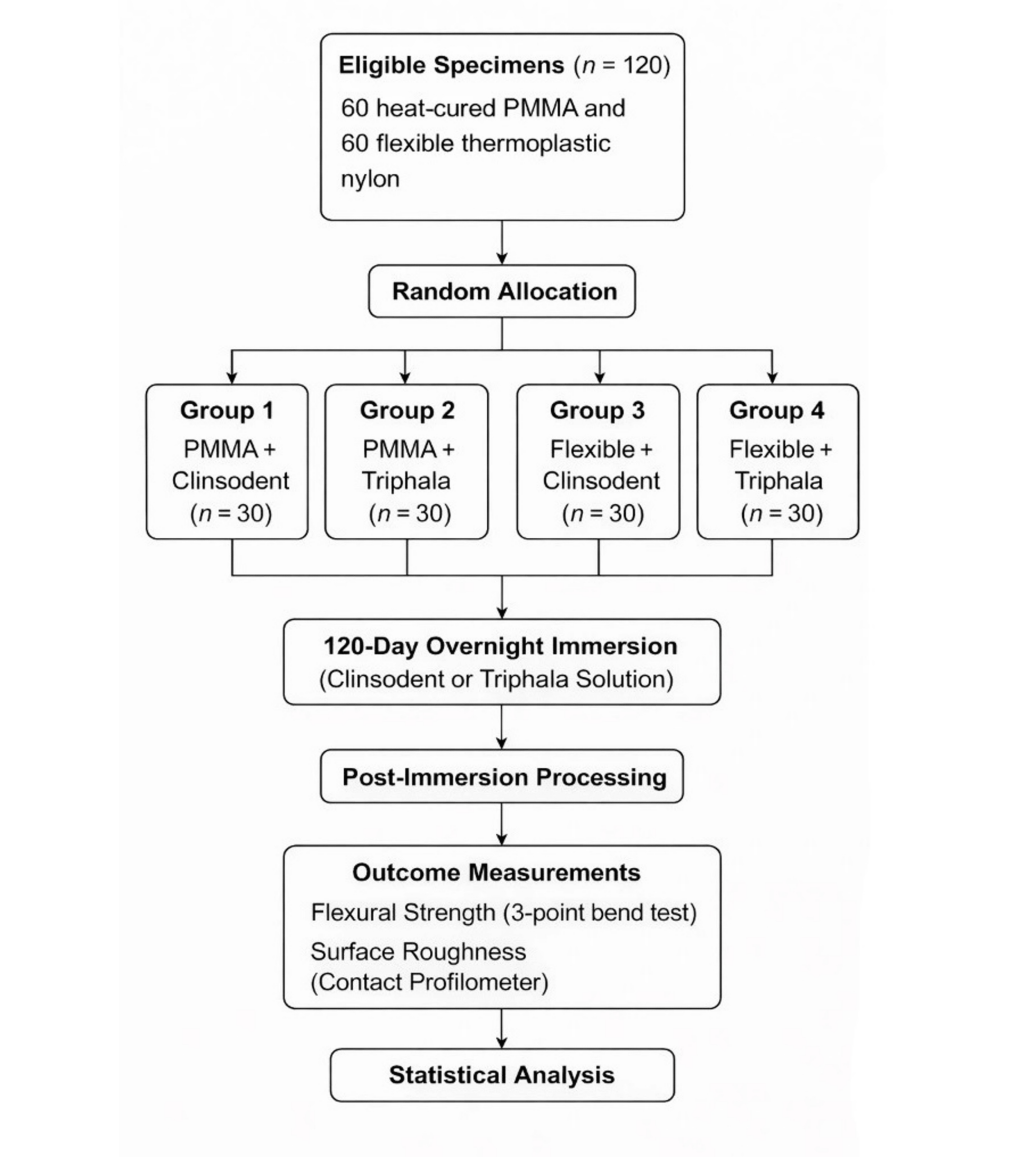
Study flow diagram PMMA, polymethyl methacrylate

The flowchart (Figure 4) illustrates the complete sequence from specimen allocation to outcome assessment.

## Results

Descriptive statistics

Descriptive statistics for flexural strength and surface roughness measured for each material and cleanser combination are presented in Table 1. The mean, SD, and SE were calculated for each combination.

**Table 1:** Descriptive statistics for flexural strength and surface roughness PMMA, polymethyl methacrylate; SE, standard error

Feature	Flexural strength (MPa)			Surface roughness, Ra (µm)		
	Mean	SD	SE	Mean	SD	SE
PMMA in commercial denture cleanser	72.4	0.1238	0.0110	0.349	0.0238	0.00434
PMMA in Triphala	91.8	0.0601	0.0282	0.239	0.0238	0.00434
Flexible in commercial denture cleanser	28.5	0.1543	0.0291	1.728	0.0413	0.00754
Flexible in Triphala	36.5	0.1594	0.0211	1.361	0.0478	0.00873

PMMA in Triphala had the highest mean flexural strength, followed by PMMA in commercial denture cleanser, flexible resin in Triphala, and flexible resin in commercial denture cleanser. For surface roughness, PMMA in Triphala exhibited the lowest mean value, indicating a smoother surface, while flexible resin in commercial denture cleanser showed the highest surface roughness. 

**Figure 5:**
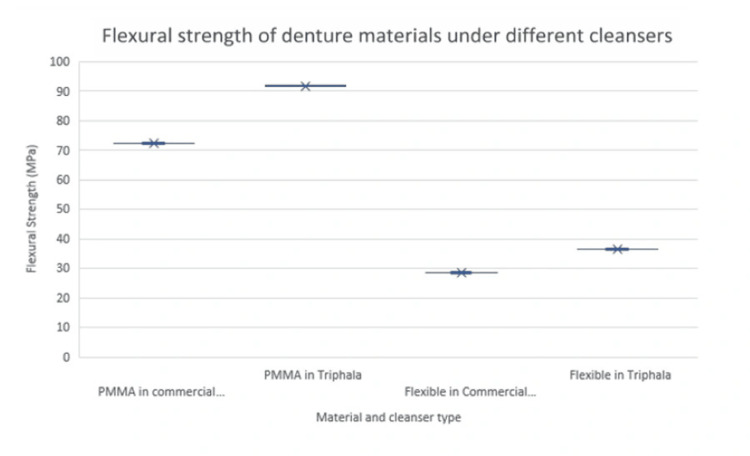
Flexural strength (MPa) of denture base materials immersed in different cleansers PMMA, polymethyl methacrylate

Figure 5 illustrates the mean flexural strength (MPa) of two denture base materials, PMMA and flexible resin, after immersion in different cleansers. PMMA samples exhibited higher flexural strength compared to flexible resin under both cleanser conditions, with Triphala-treated groups showing a noticeable increase in strength.

**Figure 6:**
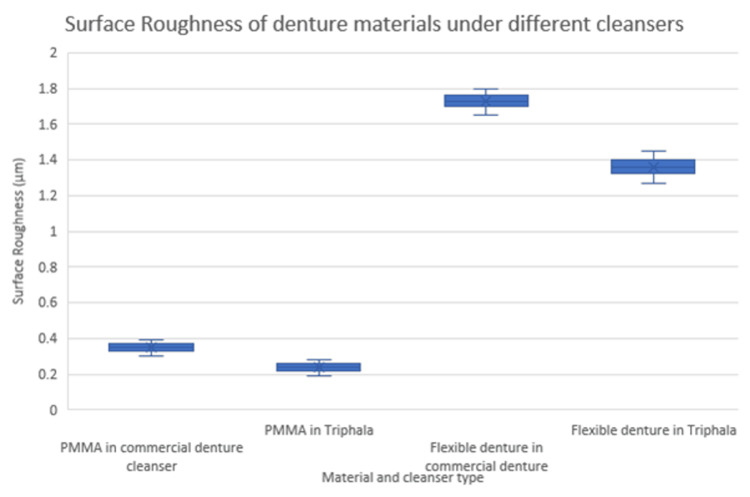
Surface roughness (Ra, µm) of denture materials across material and cleanser types PMMA, polymethyl methacrylate

Figure 6 illustrates the distribution of surface roughness values for PMMA and flexible denture materials immersed in commercial denture cleanser and Triphala. The median, interquartile range, and mean (×) are shown. PMMA in Triphala exhibited the lowest surface roughness, whereas flexible resin in commercial denture cleanser showed the highest.

Normality and homogeneity of variances

The Shapiro-Wilk test was performed to assess the normality of all features for both flexural strength and surface roughness. As shown in Table 2, all features are normally distributed.

**Table 2:** Shapiro-Wilk test for normality All variables show p>0.05, indicating no significant deviation from normality. W, Shapiro-Wilk test statistic; p, significance level; PMMA, polymethyl methacrylate

Feature	Flexural strength (MPa)			Surface roughness (µm)	
	Shapiro-Wilk			Shapiro-Wilk	
	W	p		W	p
PMMA in commercial denture cleanser	0.986		0.950	0.964	0.398
PMMA in Triphala	0.975		0.693	0.964	0.398
Flexible in commercial denture cleanser	0.985		0.939	0.974	0.639
Flexible in Triphala	0.988		0.981	0.0478	0.917

Levene’s test was conducted to evaluate the assumption of equal variances across the four groups. As shown in Table 3, the results were significant for both flexural strength and surface roughness with F(3,115)=7.12, p<0.001 and F(3,116)=7.73, p<0.001, respectively. These results indicate that there is a difference in variances across groups, suggesting that the assumption of equal variances was not met. Consequently, Welch’s ANOVA, which is robust to unequal variance, was used to identify differences among the groups.

**Table 3:** Levene's test for homogeneity of variances A significant Levene's test (p<0.05) indicates that the assumption of homogeneity of variances was violated.

	F	df_1_	df_2_	p
Flexural strength (Mpa)	7.12	3	115	<0.001
Surface roughness (µm)	7.73	3	116	<0.001

Group comparisons using Welch’s one-way ANOVA

A Welch’s one-way ANOVA was conducted to compare the effect of cleanser type and material on both flexural strength and surface roughness. The analysis revealed a significant difference among groups for both parameters, indicating that at least one group mean differed significantly from the others (Table 4).

**Table 4:** Welch’s one-way ANOVA results Welch's ANOVA was conducted due to violation of the homogeneity of variances assumption (Levene's test, p<0.001). The result indicates a statistically significant difference among group means.

	F	df_1_	df_2_	p
Flexural strength (MPa)	2.21×10⁶	3	58.4	<0.001
Surface roughness (µm)	13122	3	62.3	

Pairwise comparisons: post hoc analysis

To identify specific group differences, a Games-Howell post hoc test was performed (Table 5). For flexural strength, all pairwise comparisons were statistically significant (p<0.001). The largest mean difference was observed between PMMA (Triphala) and flexible resin (commercial denture cleanser) (mean difference=63.30, p<0.001), while the smallest, though still significant, difference was found between flexible resin (commercial denture cleanser) and flexible resin (Triphala) (mean difference=-8.01, p<0.001).

**Table 5:** Games-Howell post hoc test for pairwise comparisons PMMA, polymethyl methacrylate

Comparison	Flexural strength (MPa)		Surface roughness, Ra (µm)	
	Mean difference	p	Mean difference	p
PMMA (Triphala) - flexible (commercial denture cleanser)	63.30	<0.001	1.49	<0.001
PMMA (Triphala) - flexible (Triphala)	55.29	<0.001	1.12	<0.001
PMMA (Triphala) - PMMA (commercial denture cleanser)	19.40	<0.001	-0.110	<0.001
Flexible (commercial denture cleanser) - flexible (Triphala)	-8.01	<0.001	0.367	<0.001
Flexible (commercial denture cleanser) - PMMA (commercial denture cleanser)	-43.90	<0.001	1.379	<0.001
Flexible (Triphala) - PMMA (commercial denture cleanser)	-35.90	<0.001	1.012	<0.001

For surface roughness, all comparisons were also significant (p<0.001). The highest difference occurred between PMMA (Triphala) and flexible resin (commercial denture cleanser) (mean difference=1.49 µm), whereas the smallest difference was observed between PMMA (Triphala) and PMMA (commercial denture cleanser) (mean difference=0.11 µm). 

Overall, these results indicate that both the material type and the type of cleanser have a significant effect on both flexural strength and surface roughness.

PMMA samples, particularly those treated with Triphala, exhibited the highest flexural strength and the lowest surface roughness, indicating enhanced durability and smoother surfaces. Conversely, flexible resin samples immersed in commercial denture cleanser showed the lowest flexural strength and highest surface roughness, suggesting greater material degradation.

## Discussion

The present study revealed that both the type of denture base material and the type of cleansing agent significantly influenced flexural strength and surface roughness after prolonged immersion. As anticipated, PMMA specimens demonstrated higher flexural strength than flexible thermoplastic specimens, irrespective of the cleanser used, which aligns with findings by Gad et al. and Shah et al., who reported superior mechanical resilience of PMMA compared to flexible resins following cleanser exposure [7,13].

Effect of Triphala on flexural strength

PMMA samples immersed in Triphala exhibited the highest flexural strength, whereas those immersed in Clinsodent demonstrated a measurable reduction. This may be attributed to the mildly acidic and antioxidant nature of Triphala, which minimizes oxidative degradation of the polymer matrix [8,9]. Conversely, commercial cleansers containing strong oxidizing agents such as sodium perborate can disrupt polymer cross-linking and increase water sorption, thereby reducing mechanical stability [5,7].

Flexible resins displayed reduced flexural strength across all conditions, consistent with reports that polyamide materials are more hydrophilic and exhibit plasticizer leaching when exposed to aqueous or oxidizing agents [14,15]. Hamanaka et al. [15] demonstrated that prolonged water sorption in thermoplastic resins leads to a decrease in the modulus of elasticity and increased deformation. Thus, the superior flexural strength observed in PMMA reflects its relatively rigid polymeric structure and lower susceptibility to hydrolytic degradation.

Effect of cleansers on surface roughness

Surface roughness plays a crucial role in microbial adhesion and plaque formation, influencing the onset of denture-related mucosal inflammation [3,18,19]. In this study, Triphala-treated specimens showed lower surface roughness compared to those exposed to Clinsodent in both PMMA and flexible materials. This finding is in agreement with Sushma et al. [10], who demonstrated that herbal cleansers, including Triphala, produced smoother surfaces than chemical agents. The presence of tannins and phenolic compounds in Triphala may aid in gently removing debris and biofilm without etching the resin surface [12].

The increased roughness observed in Clinsodent-treated specimens may be attributed to oxidative breakdown of surface polymers and micro-pitting due to alkaline perborate activity [5,7]. Clinically, a smoother denture surface is desirable as it reduces bacterial colonization, improves hygiene, and minimizes the risk of denture stomatitis, a common inflammatory condition among complete denture wearers [1,3,18].

Mechanistic and biochemical considerations

The cleansing mechanism of Triphala may be linked to its polyphenolic and antioxidant constituents that chelate metal ions, disrupt microbial adhesion, and stabilize polymeric surfaces [9,12]. Its mild surfactant-like activity helps loosen organic debris without altering polymer chemistry. These features differentiate it from peroxide-based cleansers that release reactive oxygen species, which can induce oxidative stress and material degradation over time [5,7].

Clinical implications

From a clinical standpoint, the use of Triphala as a denture cleanser could be advantageous for geriatric patients and those in resource-limited settings where commercial cleansers are costly or unavailable. Triphala's biocompatibility, accessibility, and minimal environmental impact position it as a sustainable alternative to chemical cleansers. Maintaining lower surface roughness and adequate flexural strength can help extend denture lifespan while minimizing microbial colonization.

Limitations and future directions 

This study was conducted under controlled in vitro conditions, which do not fully replicate the dynamic oral environment, including the effects of salivary enzymes, pH fluctuations, and mechanical stresses. Therefore, the results may have limited direct clinical correlation. Additionally, only two cleansing agents were evaluated, which restricts the generalizability of the findings. Future in vivo studies are warranted to validate these results, assess additional parameters such as color stability, microbial load reduction, and patient-reported outcomes, and determine the optimal concentration and immersion time of Triphala for standardized clinical protocols.

## Conclusions

Triphala demonstrated effective cleansing properties, improved surface roughness, and maintained acceptable flexural strength in both PMMA and flexible denture base materials after 120 days of overnight immersion. These findings suggest that Triphala is a safe, economical, and accessible alternative to commercial denture cleansers, especially in resource-limited settings. Triphala is a promising candidate warranting further trials with thermal cycling, microbiological endpoints, and in vivo testing to confirm its clinical applicability.
